# Editorial: Fighting Antimicrobial Resistant Microorganisms: Current Status and Emerging Strategies Using Nanomaterials

**DOI:** 10.3389/fbioe.2021.764664

**Published:** 2021-09-28

**Authors:** María Gabriela Paraje, Paulina Laura Páez

**Affiliations:** ^1^ Instituto Multidisciplinario de Biología Vegetal (IMBIV), Consejo Nacional de Investigaciones Científicas y Técnicas (CONICET), Córdoba, Argentina; ^2^ Cátedra de Microbiología, Facultad de Ciencias Exactas, Físicas y Naturales, Universidad Nacional de Córdoba, Córdoba, Argentina; ^3^ Unidad de Investigación y Desarrollo en Tecnología Farmacéutica (UNITEFA), Consejo Nacional de Investigaciones Científicas y Técnicas (CONICET), Córdoba, Argentina; ^4^ Departamento de Ciencias Farmacéuticas, Facultad de Ciencias Químicas, Universidad Nacional de Córdoba, Córdoba, Argentina

**Keywords:** biogenic nanoparticles, antimicrobial agents, bacterial infection, multidrug-resistant strains, photodynamic therapy

Antimicrobial resistance is a global crisis and, along with the availability of few effective antimicrobials, emphasize the need for new approaches and different safe and effective alternatives to current antimicrobials strategies. Recent progress in biomedical applications has been proposed to prevent or treat resistant pathogens due to the limited efficacy of antimicrobial agents. Metal nanoparticles (NPs) are attracting huge interest in nanobiology and nanomedicine fields as a new class of antimicrobial agents, because of their ease of preparation, their high stability over time, and lower probability of developing resistance by microorganisms. In the past 2 decades, NPs have received attention as a potential future treatment of a wide range of serious infections. Particularly metal NPs are considered promising due to their unique physicochemical properties, chemical composition, size, surface charge, and similar size to intra- and extra-cellular components that allow specifically interact with molecular and sub-cellular structures. Furthermore, the release of metal ions from these NPs also triggers the death of microbial pathogens. For example, most metal-based nanomaterials can generate broad-spectrum and potent antimicrobial activity by specific nano-biological effects including physical damage as well as cellular stress to inactivate microbial cells. In this sense, a disturbance in the prooxidant-antioxidant balance can lead to damage to carbohydrates, lipids, proteins, and nucleic acids and, if this damage is not repaired, cell death can occur (Quinteros et al., 2018).

This Research Topic focused on “*Fighting Antimicrobial Resistant Microorganisms: Current Status and Emerging Strategies Using Nanomaterials*” within the scope of the article series “Bioengineering and Biotechnology” featured by the open-access journal “Frontiers” outstanding examples of emerging strategies using NPs in the treatment of a wide range of serious infections. Two important basic articles included in this research topic are written in the form of original research using biogenic silver nanoparticles (AgNps) against antibiotic-resistant pathogens. One of these articles highlights a simple and eco-friendly method for the biosynthesis of AgNps using *Lysinibacillus xylanilyticus* strain MAHUQ-40, and was used to investigate their antibacterial activity and mechanisms against *Vibrio parahaemolyticus* and *Salmonella typhimurium*. Structural and morphological changes and damage to membrane integrity were observed by field emission-transmission electron microscopy (FESEM) (Md. Amdadul Huq).

The second original research included in this volume was performed with the aim of knowing the extracellular production of AgNps using fungal extracts. The evaluation of their antimicrobial activity against resistant and multi-resistant *S. typhimurium* strains suggested that the NPs produced damage in several bacterial cell structures. It was studied through Atomic Force Microscopy (AFM) and Raman Confocal Microscopy, as an approach to advance in the understanding of the antimicrobial mechanism of the biogenic NPs against multi-resistant bacteria (María Belén Estevez et al.).

The subsequent publication has focused on publishing the synergistic antibacterial and cell surface topology study of carbon nanodots (C-dot) and tetracycline against *Escherichia coli*. The authors showed cell damage and binding of C-dot with the bacterial cell membrane by Scanning Electron Microscopy and validated the topological changes, cell surface roughness, and significant change in height profile under AFM (Dhermendra K. Tiwari et al.).

Finally, this volume included a mini-review highlighting the recent research advances covering scientific approaches to the photosensitization process and supramolecular chemistry applied to the development of efficient applications of antimicrobial photodynamic therapy, with a brief discussion of the remaining challenges to further research for new antimicrobial photodynamic treatments (Cecilia Vera et al.).

Nanotechnology offers the opportunity to exploit antimicrobial properties by manipulating their size to dimensions on the nanometer scale. The importance to eradicate microbial resistance to multiple antimicrobials justifies the need to find new antimicrobial drugs in the near future. In conclusion, we are proud to present our Research Topic “Fighting Antimicrobial Resistant Microorganisms: Current Status and Emerging Strategies Using Nanomaterials” in Frontiers in Bioengineering and Biotechnology. We believe that all readers should experience the possibilities surrounding biotechnological tools as well as in the way to manipulate bio-nano interactions for medical applications to combat resistant microorganisms as has been showed in our topic. We are also optimistic that this Research Topic provides outstanding insights into the important issues of nanomaterials associated with the eradication and control of infections. Understanding the interactions between microorganisms by specific nano-biological effects and NPs play a crucial role in the development of the biomedical application of nanomedicine.
